# Novel biomarkers predict liver fibrosis in hepatitis C patients: alpha 2 macroglobulin, vitamin D binding protein and apolipoprotein AI

**DOI:** 10.1186/1423-0127-17-58

**Published:** 2010-07-15

**Authors:** Ai-Sheng Ho, Chun-Chia Cheng, Shui-Cheng Lee, Meng-Lun Liu, Jing-Ying Lee, Wen-Ming Wang, Chia-Chi Wang

**Affiliations:** 1 Division of Gastroenterology, Cheng Hsin General Hospital, Taipei, Taiwan; 2 Graduate Institute of Medical Sciences, College of Medicine, Taipei Medical University, Taipei, Taiwan; 3 Institute of Nuclear Energy Research, Atomic Energy Council, Taoyuan, Taiwan; 4 Division of Internal Medicine, Kaohsiung Municipal Hsiao-Kang Hospital, Kaohsiung Medical University, Kaohsiung, Taiwan; 5 Division of Gastroenterology, Department of Internal Medicine, Kaohsiung Medical University Hospital, Kaohsiung, Taiwan; 6 Department of Medicine, Faculty of Medicine, College of Medicine, Kaohsiung Medical University, Kaohsiung, Taiwan; 7 Division of Gastroenterology, Buddhist Tzu Chi General Hospital, Taipei branch, Taiwan

## Abstract

**Background:**

The gold standard of assessing liver fibrosis is liver biopsy, which is invasive and not without risk. Therefore, searching for noninvasive serologic biomarkers for liver fibrosis is an importantly clinical issue.

**Methods:**

A total of 16 healthy volunteers and 45 patients with chronic hepatitis C virus (HCV) were enrolled (F0: n = 16, F1: n = 7, F2: n = 17, F3: n = 8 and F4: n = 13, according to the METAVIR classification). Three serum samples of each fibrotic stage were analyzed by two-dimension difference gel electrophoresis (2D-DIGE). The differential proteins were identified by the cooperation of MALDI-TOF/TOF and MASCOT; then western blotting and Bio-Plex Suspension Array were used to quantify the protein levels.

**Results:**

Three prominent candidate biomarkers were identified: alpha 2 macroglobulin (A2M) is up regulated; vitamin D binding protein (VDBP) and apolipoprotein AI (ApoAI) are down regulated. The serum concentration of A2M was significantly different among normal, mild (F1/F2) and advanced fibrosis (F3/F4) (*p *< 0.01). The protein levels of VDBP and ApoAI were significantly higher in normal/mild fibrosis, when compared to those in advanced fibrosis (both *p *< 0.01).

**Conclusions:**

This study not only reveals three putative biomarkers of liver fibrosis (A2M, VDBP and ApoAI) but also proves the differential expressions of those markers in different stages of fibrosis. We expect that combination of these novel biomarkers could be applied clinically to predict the stage of liver fibrosis without the need of liver biopsy.

## Introduction

In hepatitis C virus (HCV)-infected patients, liver fibrosis is a noticeable disease and could progress to liver cirrhosis or hepatocellular carcinoma gradually. Although the pathogenesis of HCV-infected fibrosis is poorly understood, liver fibrosis may be a response of repair when liver is injured or inflamed [1]. In addition, the detection of early stage of liver fibrosis is very important before the irreversible damage occurs. Liver biopsy followed by histological examination is still the gold standard for the assessment of liver fibrosis [2]. However, it has several limits and disadvantages such as invasive character and sampling error [3-5]. Therefore, it is necessary to have a reliable and noninvasive assessment for liver fibrosis.

Two-dimension difference gel electrophoresis (2D-DIGE), first proposed by Unlu et al [6], allow different samples to be labeled with cy3 or cy5 flour in one gel. It used cy2-labeled internal standard to tell the differences and found out the reliable biomarkers among different gels. Furthermore, multi-analyte profiling (xMAP) technique was used to quantify the concentration of putative biomarkers. By using these methods, we could not only identify but also quantify the candidate biomarkers, which could be a serologic predictor for the severity of liver fibrosis. In this study, we enrolled patients of chronic HCV infection and healthy controls, and used proteomic technique to analyze their sera. The aim is to search for noninvasive serological biomarkers of liver fibrosis, which could predict the stage of fibrosis without the need of liver biopsy.

## Materials and methods

### Serum samples

Totally 61 serum samples from 45 patients of chronic HCV infection and 16 healthy volunteers were obtained from Cheng Hsin General Hospital in Taiwan (approval No. 97016). The assessment of liver fibrosis was based on liver biopsy and subsequent histological examination. The stage was determined according to METAVIR classification [7]. The stages of liver fibrosis were distributed as following in the chronic hepatitis C patients: F1, n = 7, F2, n = 17, F3, n = 8 and F4, n = 13. The serum samples of healthy control (F0 stage, n = 16) were obtained from healthy volunteers who had no evidence of known hepatitis. Liver biopsy was not performed in these subjects due to ethical issues.

### Two dimension-difference gel electrophoresis

The serum samples were diluted 1:5 with lysis buffer (50 mM Tris-HCl, 8M urea, 4% (w/v) 3-[(3-Cholamidopropyl) dimethylammonio]-1-propanesulfonate, and pH 8.5). The protein concentration was measured (DC™ Protein Assay Kit, Bio-Rad) and individual 50 μg of protein sample was allowed to label with 400 pmol of cy3 or cy5. In addition, pooled internal standard (400 μg) was allowed to label with 3200 pmol of cy2. Subsequently the solution was added 1 μL of 10 mM lysine to stop the reaction. The serum samples of labeled-cydyes and its arrangement are presented in Table 1. Each mixture was added twofold volume of sample buffer (8 M urea, 20 mM dithiothreitol, 4% (w/v) 3-[(3-Cholamidopropyl) dimethylammonio]-1-propanesulfonate, 0.5% (v/v) IPG buffer and few bromophenol blue) and performed with 18 cm, pH 4-7 IPG strips for the isoelectric focusing (IEF) at 20°C (30000 Vh) (IPGphor system, GE Healthcare). After equilibration, the strips were overlaid on individual 12.5% polyacrylamide gels and added 0.5% agarose to immobile the strips. After electrophoresis, the cy2, cy3, and cy5-labeled images were acquired (Typhoon TRIO Variable Mode Imager, GE Healthcare) using 488, 532, and 633 nm lasers with an emission filter of 520, 532, and 670 nm respectively. All gels were analyzed by using DeCyder 6.5 software (GE Healthcare) to select and match all protein spots. The estimated number of spots was set at 10000. Spot maps of the filtered gels were saved and imported to Biological Variation Analysis program for inter-gel matching and statistical analyses. The interesting protein spots were selected according to one-way ANOVA with a significant value of 0.05 or less.

**Table 1:** Arrangement for protein samples labeled with three CyDye flours

Gel	Cy2	Cy3	Cy5
1	Pool of samples	F0 (1)	F2 (1)
2	Pool of samples	F3 (1)	F0 (2)
3	Pool of samples	F0 (3)	F4 (1)
4	Pool of samples	F2 (2)	F1 (1)
5	Pool of samples	F1 (2)	F3 (2)
6	Pool of samples	F4 (2)	F1 (3)
7	Pool of samples	F3 (3)	F2 (3)
8	Pool of samples	F0 (1)	F4 (3)

### In-gel tryptic digestion

The gels were stained with Sybro Ruby (sigma) for at least four hours and then destained with 10% methanol/7% acetic acid for exactly 30 min. The interesting proteins in the gels were picked up manually on UV transilluminator (Spectroline). Those gel particles were washed with 10% methanol/7% acetic acid overnight to remove Sybro Ruby chemicals thoroughly; 100 μL of 25 mM ammonium bicarbonate in 50% acetonitrile for 15 min; 200 μL of 25 mM ammonium bicarbonate in deionized water for 15 min twice. The saturated gel particles were added enough acetonitrile to shrink for 5 min. After drying down, the gel particles were added 3 μL of 20 ng/μL trypsin in 25 mM ammonium bicarbonate at 4°C for 1 hour and subsequently added 3 μL of 25 mM ammonium bicarbonate to keep the gels wet at 56°C for 1 hour. After In-gel digestion the solution were added 2 μL of 100% acetonitrile with 1% trifluoracetic acid and sonicated for 10 min to release peptides from gel particles.

### Mass spectrometric analysis for protein identification

Each trypsin-digested solution was mixed 1:1 with 10 mg/mL α-cyano-4-hydroxycinnamic acid in 50% acetonitrile/0.1% trifluoracetic acid and spotted on AnchorChip MALDI target (Bruker Daltonics GmbH, Bremen, Germany). Peptides were analyzed with MALDI-TOF/TOF UltraflexIII (Bruker Daltonics) by peptide mass fingerprinting after calibration in positive reflection mode under 20 KV and calculated the molecular weight with FlexAnalysis™ 3.0 software (Bruker Daltonics). MASCOT 2.2 (Matrix Science) was used to match the peptides with NCBI or Swiss-Prot database for protein identification. The calculation was restricted to human taxonomy, allowing carbamidomethyl cysteine as a fixed modification and oxidized methionine as a variable modification. The probability was based on Mowse score calculated from -10 × Log (P), where P was the probability that the observed match was a random event. Protein scores greater than 56 were significant (*p *< 0.05). Moreover, one of the major peptide peaks appeared on the spectrum was used to confirm the searching result by peptide fragment fingerprinting method.

### Western blotting

Each serum sample was diluted 1:10 with sodium dodecyl sulfate buffer (50 mM Tris-Cl, 8 M urea, 30% glycerol, 2% sodium dodecyl sulfate, 20 mM dithiothreitol and 0.1% bromophenol blue). The sample solutions were heated at 100°C for 5 min; and then 2 μL of samples (approximately 20-30 μg) were loaded to 4-12% sodium dodecyl sulfate-polyacrylamide gel electrophoresis (SDS-PAGE, Invitrogen). The iblot (Invitrogen) was used for transforming the proteins to polyvinylidene fluoride (PVDF). After using 0.5% milk to blot the PVDF for 30 min, the A2M, ApoAI and VDBP were detected by rabbit anti-A2M antibody (AbD serotec), chicken anti-ApoAI antibody (CHEMICON) and rabbit anti-VDBP antibody (AbD serotec) for two hour at room temperature. After washing three times in PBS buffer (10 mM sodium phosphate, pH7.4 and 0.9% sodium chloride), the second antibody conjugated with horseradish peroxidase was added to incubate for one hour. The ECL detection system (Millipore) was used and the images were acquired by Imaging System (Gel Doc XR System, Bio-Rad) depending on the moderate exploring time.

### Bio-plex suspension array system

Bio-Plex 200 Suspension Array System (Bio-Rad) was based on flexible multi-analyte profiling (xMAP) technique developed by Luminex Corporation using the principle of sandwich immunoassay. Individual serum sample was diluted 100 thousands fold with Bio-Plex human serum diluent. For A2M measurement, Bio-Plex Pro Human Acute Phase 4-Plex Panel (cat. 171-A4009M, Bio-Rad) was performed. For ApoAI and VDBP measurement, there are many processes need to complete, including antibody labeled with microsphere and biotin individually. These experiments were completed by following the manual of Amine Coupling Kit (Bio-Rad) and Lynx Rapid Biotin Antibody Conjugation Kit (AbD Serotec) respectively. After pre-wet of 96-well plate, each 50 μL of diluted sample was incubated with 1.25 × 10^6 ^microspheres, which labeled with primary antibody, at 300 rpm for exactly 1 hour. Subsequently each 50 μL of biotin-conjugated secondary antibody (2 μg/mL) was added to incubate at 300 rpm for 30 min. Finally 50 μL of streptavidin-Phycoerythrin was added to each well and mixed at 300 rpm for 10 min. After drying up and washing, 125 μL of assay buffer was allowed to suspend each well of microspheres. The protein concentration was measured by Bio-Plex 200 Suspension Array System. SPSS software (Ver.14.0; SPSS Inc.) was used to calculate the *p *value and to present the curve of expressional trend with Box-and-Whisker Plot.

## Results

The serum samples labeled with cy2, cy3 or cy5 individually are presented in Table 1. From F0 to F4 fibrotic stages, we selected three samples of each stage to do 2D-DIGE experiments. For this purpose, 15 samples needed to separate into eight gels, in which F0-1 was used in gel 1 and gel 8 for fitting the arranging design. This design would not influence the data calculation. In this study we did not use albumin/IgG removal kit to remove high abundant albumin and IgG because we wanted to simplify the experimental process. Moreover removing albumin protein would remove albumin-binding proteins in the same time and influence the reproducibility.

**Figure 1:**
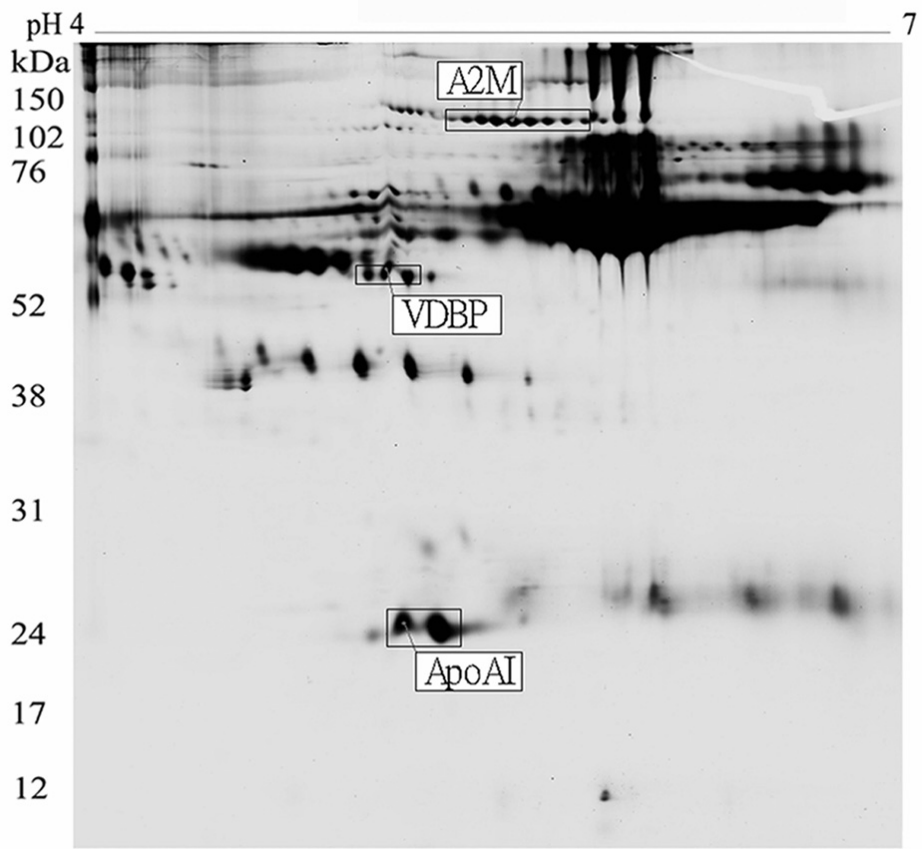
**Three novel biomarkers of liver fibrosis, A2M, VDBP and ApoAI, appear on the location of 162 kDa, 52 kDa and 28 kDa in the 2D-DIGE gels**. Observably there are several adjacent spots near A2M, VDBP and ApoAI protein; the adjacent spots were identified as same as A2M, VDBP or ApoAI respectively.

**Figure 2:**
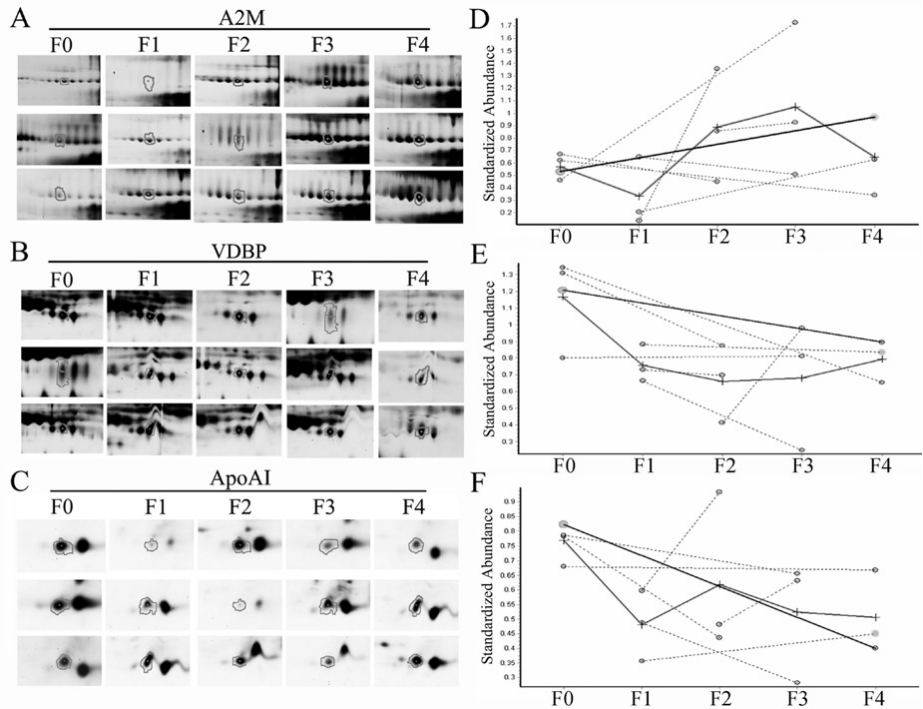
**The protein expressions of A2M (A), VDBP (B) and ApoAI (C) are presented in eight gels**. The expressional trends of A2M (D), VDBP (E) and ApoAI (F) were calculated with DeCyder 6.5 software. A2M is an up-regulated protein; VDBP and ApoAI are down-regulated proteins with hepatic fibrosis development.

After the protein matching and statistics calculation with DeCyder 6.5 software, there were three putative proteins selected (*p *≤ 0.05). These protein locations in 2D-PAGE gel are shown in Fig. 1. The three putative proteins were found out that they all appeared in eight gels (Fig. 2A, Fig. 2B and Fig. 2C). The three proteins were excised from gels, digested with trypsin, and identified by the cooperation of MALDI-TOF/TOF with MASCOT software (link to NCBI database, http://www.ncbi.nlm.nih.gov/). Alpha 2 macroglobulin (A2M) is up regulated whereas vitamin D binding protein (VDBP) and apolipoprotein AI (ApoAI) are down regulated in hepatic fibrosis serum (Fig. 2D, Fig. 2E and Fig. 2F; Table 2). Meanwhile, we noticed that A2M protein had a series of adjacent spots appeared in 2D-PAGE; besides, VDBP and ApoAI had two and one adjacent spots respectively (Fig. 1). Those different spots were identified as the same results as A2M, VDBP or ApoAI respectively.

**Table 2:** Protein spots identified by MALDI/TOF-TOF-MS

**Protein**^**§**^	GenBank	coverage ratio	regulation	***p *value**^**¶**^
A2M	gi:224053	29%	↑	0.015
VDBP	gi:18655424	38%	↓	0.020
ApoAI	gi:90109664	49%	↓	0.027

^§^A2M, alpha 2 macroglobulin; VDBP, vitamin D binding protein; ApoAI, apolipoprotien AI.^¶^The *p *value was calculated by one way ANOVA with DeCyder 6.5 software. ↑, present in normal and each fibrotic stage but the protein expression in fibrotic stages (F1-F4) is higher than that in normal control (F0); ↓, present in normal and each fibrotic stage but the protein expression in moderate/advanced fibrotic stages (F3/F4) is lower than that in normal control/mild fibrotic stage (F0/F1).

**Figure 3:**
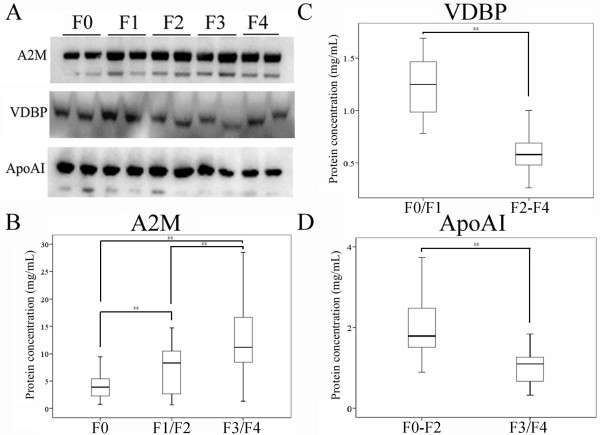
**Verification of A2M, VDBP and ApoAI by western blotting (A) and quantification of A2M (B), VDBP (C) and ApoAI (D) by Bio-Plex Suspension Array System**. A2M is increased in the F1-F4 stages; VDBP is decreased in the F2-F4 stage and ApoAI is decreased in the F3/F4 stages. **p < 0.01.

Although 2D-DIGE analyses already demonstrated that the protein levels and expressional trends for each candidate biomarkers were apparently distinct in fibrotic stages, using antibody to verify the result was important. We selected two samples of each fibrotic stage to analyze the protein expressions by using western blotting for verifying the protein identification. A2M was detected having higher protein expression in F1-F4 stage than in F0 stage (Fig. 3A). Moreover, VDBP and ApoAI were down regulated (Fig. 3A). Particularly the protein expression of VDBP from mild fibrosis (F0/F1) to advanced fibrosis (F2-F4) was decreased (Fig. 3A and Fig. 3C). The protein expression of ApoAI was changeable only in F3/F4 compared with that in F0-F2 stage (Fig. 3A and Fig. 3D).

We used Bio-Plex Suspension Array System to measure the absolute protein concentration of A2M, VDBP and ApoAI according to protein standard curve. We found that the serum concentration of A2M from F0 to F4 was increased significantly (F0: 4.3 ± 2.8, F1/F2: 7.2 ± 4.3, F3/F4: 13.0 ± 6.8 mg/mL, *p *< 0.01) (Fig. 3B). The correlation coefficient of A2M was 0.98. The result suggests that A2M protein could distinguish the stages among normal (F0), mild (F1/F2) and advanced fibrosis (F3/F4). The serum concentration of VDBP was decreased from F0/F1 to F2-F4 stage (F0/F1: 1.2 ± 0.3 mg/mL, F2-F4: 0.6 ± 0.2 mg/mL, *p *< 0.01) (Fig. 3C). The result indicates that VDBP protein could differentiate F0/F1 from F2-F4 stage. The protein concentration of ApoAI was decreased in F3/F4 stage (F0-F2: 2.0 ± 0.7 mg/mL, F3/F4: 1.1 ± 0.5 mg/mL, *p *< 0.01) (Fig. 3D). This result implies that ApoAI could be a biomarker to differentiate normal/mild (F0-F2) from advanced fibrosis (F3/F4).

## Discussion

Searching for novel serological biomarkers of HCV-infected fibrosis is to avoid the use of invasive liver biopsy. Developing an efficient and noninvasive method for liver fibrosis is important for prognosis and treatment plan in patients with chronic hepatitis C virus. A noninvasive diagnosis of liver fibrosis could also enhance the development of antifibrotic therapies. There are several non-invasive methods to assess liver fibrosis in patients with chronic hepatitis C, including FibroScan (FS) [8,9], Fibrotest (FT) [10]. However, FS could not assess liver fibrosis properly when patients were overweight or morbid obese; besides, stiffness measurement is hard to acquire in ascitic patients [8]. Ziol M et al. [11] indicated that FS appeared as a reliable tool to detect significant fibrosis or cirrhosis rather than early liver fibrosis. Castera L et al. [12] suggested that the combined use of FS and FT to assess liver fibrosis could avoid liver biopsy in most patients with chronic hepatitis C. In our study, although two of the three identified biomarkers, A2M and ApoAI, are the same as that in FT, ApoAI is decreased significantly only in advanced fibrosis (F3/F4). This result suggests ApoAI could only be an indictor for advanced fibrosis or cirrhosis. In addition, this is the first report that VDBP could be a biomarker of liver fibrosis in patients with chronic hepatitis C.

A2M is a well-known biomarker of hepatic fibrosis [13] and a significant component of measuring liver fibrosis in FibroTest, FIBROSpect II, Fibrometer or Hepascore [10,14-16]. A2M is able to inactivate an enormous variety of proteinases and inhibit fibrinolysis by reducing plasmin and kallikrein. In inflammatory or injured liver, the increase of A2M inhibit catabolism of matrix proteins and thus cause liver fibrosis [17-20]. Gangadharan B et al. indicated that thioester cleavage of A2M may increase gradually with the development of fibrosis [21]. In our study, we consistently confirmed that serum concentration of A2M may be an indicator to predict liver fibrosis. However, serum A2M level is increased in patients with depression or nephrotic syndrome [22,23]. Therefore, while using A2M as liver fibrosis biomarker, other biomarkers are needed to decrease the interference of other diseases such as nephrotic syndrome or depression.

VDBP is also known as Gc-globulin (group-specific component globulin) which binds and transports vitamin D metabolites [24,25]. The significant function of VDBP is involved in actin scavenger system, thus to protect the organism from the toxic effect of intravascular actin polymerization [26]. Moreover, VDBP can also be converted into a macrophage-activator factor, and actin-free VDBP is associated with organ dysfunction in acute liver failure [27,28]. However, this is the first study to validate that the level of VDBP is negatively associated with the development of liver fibrosis. ApoAI, applied also in FibroTest and Fibrometer test [29,30], was a putative biomarker of HCV-infected fibrosis which was also verified to be a protein with down regulation in liver hepatic fibrosis in this study. Moreover, we found that the level of ApoAI was changeable between F0-F2 and F3/F4 stage. Our result demonstrates that ApoAI is a candidate biomarker of advanced fibrosis (F3/F4).

2D-DIGE technologies, a useful tool in proteomics analysis recently, were used to search for reliable biomarker of liver fibrosis in our study. Cy2-labeled internal standard can correct the analytical error among gels so that more than two samples can be analyzed. Furthermore, the samples, which were labeled with high sensitive CyDye, could pour together in one gel. Thus, thousands of serum proteins could be easily analyzed and identified as reliable biomarkers [31,32]. In addition, there were several advantages for 2D-DIGE technique such as reducing variation among gels and increasing the reproducibility of proteins [33]. The sensitivity of CyDye flours (<0.05 ng) was better than silver staining or coomassine blue staining [34,35]. Therefore 2D-DIGE was more powerful than traditional two-dimension electrophoresis (2DE) in biomarker discovery. However, it still had some limitations in detecting the hydrophobic proteins, proteins bigger than 200 kDa, or those smaller than 10 kDa. Many proteins of extreme acidity or basicity were also not presented in the gels [36]. Moreover, because CyDye flours needed to conjugate with lysine residue of proteins, the high abundant proteins with few or no lysine residues were difficult to be detected.

The mean in distinct stages was used as cut-off value to define the severity of liver fibrosis (A2M: <4.3 mg/mL, score 0; 4.3-7.2 mg/ml, score 1; 7.2-13.0 mg/ml, score 2; >13.0 mg/ml, score 3; VDBP: >1.2 mg/mL, score 0; 0.6-1.2 mg/ml, score 1; <0.6 mg/ml, score 2; ApoAI: >2.0 mg/mL, score 0; 1.1-2.0 mg/ml, score 1; <1.1 mg/ml, score 2, Table 3). In this algorithm, the combining score of three biomarkers from 0 to 3 represent normal (F0/F1); score from 4 to 7 represent liver fibrosis (F2-F4). The sensitivity and specificity are 75% and 79% respectively. Furthermore, the stages of liver fibrosis from F1 to F4 could be predicted accurately (the median of combining score: F0 = 2, F1 = 2.5, F2 = 4, F3 = 4.5, F4 = 6). As we know, the main problem of available serologic tests to predict the stage of liver fibrosis is the tiny difference between F1 and F2; F2 and F3. The addition of these biomarkers especially VDBP and ApoAI in the algorithm could be helpful to separate these two stages of liver fibrosis. Whether combining other known biomarkers of liver fibrosis such as tissue inhibitor of metalloproteinases-1 (TIMP-1), hyaluronic acid (HA), N-terminal propeptide of type III procollagen (PIIINP) or YKL-40 [30,37,38] may increase the sensitivity and specificity of this algorithm needs further studies to confirm.

**Table 3:** Cut-off value of A2M, VDBP and ApoAI to predict liver fibrosis^§^

A2M	<4.3	4.3-7.2	7.2-13.0	>13.0
Score	0	1	2	3
VDBP	>1.2	0.6-1.2	<0.6	
Score	0	1	2	

ApoAI	>2.0	1.1-2.0	<1.1	
Score	0	1	2	

^§^The measuring unit is mg/mL. In this algorithm of three biomarkers, combining score from 0 to 3 present normal (F0/F1); score from 4 to 7 present liver fibrosis (F2-F4). A2M, alpha 2 macroglobulin; VDBP, vitamin D binding protein; ApoAI, apolipoprotien AI.

In summary, this study not only reveals three putative biomarkers of liver fibrosis (A2M, VDBP and ApoAI) but also proves the differential expressions in different stages of fibrosis. Furthermore, we discovered a novel biomarker, VDBP, which is decreased in liver fibrosis (F2-F4). In addition, the algorithm combining three biomarkers could be used clinically to predict the stage of liver fibrosis and to reduce the use of liver biopsy.

## Competing interests

The authors declare that they have no competing interests.

## Authors' contributions

ASH assisted with diagnosis for patients and collection of fibrotic serum samples; CCC assisted with article writing, protein identification from the gel spots and western blotting experiment; MLL and JYL assisted with preparation for the serum samples and judgment for fibrotic stages; SCL and WMW were projective leaders and assisted with experimental design; CCW assisted with article revising. All authors read and approved the final manuscript.
